# *S. pneumoniae *transmission according to inclusion in conjugate vaccines: Bayesian analysis of a longitudinal follow-up in schools

**DOI:** 10.1186/1471-2334-6-14

**Published:** 2006-01-30

**Authors:** Simon Cauchemez, Laura Temime, Alain-Jacques Valleron, Emmanuelle Varon, Guy Thomas, Didier Guillemot, Pierre-Yves Boëlle

**Affiliations:** 1 INSERM U707, Paris, France; 2 Université Pierre et Marie Curie, Paris, France; 3 CNAM, Paris, France; 4 Assistance Publique – Hôpitaux de Paris, Paris, France; 5 Centre de Référence du Pneumocoque, Hôpital Européen George Pompidou, Paris, France; 6 Institut Pasteur, Paris, France

## Abstract

**Background:**

Recent trends of pneumococcal colonization in the United States, following the introduction of conjugate vaccination, indicate that non-vaccine serotypes tend to replace vaccine serotypes. The eventual extent of this replacement is however unknown and depends on serotype-specific carriage and transmission characteristics.

**Methods:**

Here, some of these characteristics were estimated for vaccine and non-vaccine serotypes from the follow-up of 4,488 schoolchildren in France in 2000. A Bayesian approach using Markov chain Monte Carlo data augmentation techniques was used for estimation.

**Results:**

Vaccine and non-vaccine serotypes were found to have similar characteristics: the mean duration of carriage was 23 days (95% credible interval (CI): 21, 25 days) for vaccine serotypes and 22 days (95% CI: 20, 24 days) for non-vaccine serotypes; within a school of size 100, the Secondary Attack Rate was 1.1% (95% CI: 1.0%, 1.2%) for both vaccine and non-vaccine serotypes.

**Conclusion:**

This study supports that, in 3–6 years old children, no competitive advantage exists for vaccine serotypes compared to non-vaccine serotypes. This is an argument in favour of important serotype replacement. It would be important to validate the result for infants, who are known to be the main reservoir in maintaining transmission. Overall reduction in pathogenicity should also be taken into account in forecasting the future burden of pneumococcal colonization in vaccinated populations.

## Background

Less than 10 *Streptococcus pneumoniae *(*S. pneumoniae*) serotypes, among more than 90, have been included in the *S. pneumoniae *conjugate vaccine formulation. Since these serotypes account for a large part of carriage (almost 80% of all carriage in the United States [1]) and the vaccine protects against colonization [2], a reduction in overall carriage after vaccination is expected, and has indeed been observed in vaccinated populations [3]. However, recent trends of pneumococcal colonization in the United States, following the introduction of conjugate vaccination, indicate serotype replacement [4,5], whereby a decrease in vaccine serotypes carriage is followed by an increase in carriage of non-vaccine serotypes. The eventual extent of this replacement is as yet unknown.

Mathematical models have predicted that replacement could occur in case of direct competition between serotypes in the absence of cross-immunity [6-9]. In these models, the extent of replacement is expected to be important if the degree of competition is high; and in this latter case, all the more that vaccine and non-vaccine serotypes have the same duration of carriage and transmission rate. However, this last assumption (same duration and transmission) could be challenged, considering the wide heterogeneity observed in serotype-specific prevalence, in favor of reduced capacity to colonize in the less frequent serotypes.

Ekdahl et al. [10] reported that there was no difference in the duration of carriage among 5 serogroups, 3 of which are included in the vaccine. Smith et al. [11] found that average duration of carriage ranged 6.7 to 62.5 days among 25 serotypes, 6 of which are included in the vaccine. However, these variations did not clearly identify vaccine serotypes as having longer carriage duration. Reported transmission rates of *S. pneumoniae *estimated from household data did not investigate serotype specific characteristics, nor inclusion in the vaccine [12,13]. While it has been reported that non-vaccine serotypes may be less invasive [14], there is currently little information on carriage and transmission characteristics according to serotype inclusion in the vaccine. This lack of knowledge may stem from the difficulty to analyze field data on colonization, which generally consist of samples irregularly spaced in time, and lack information about the times of colonization and decolonization.

Here, using recent computational-based statistical techniques, we analyze a prospective follow-up study of schoolchildren in France, with a view to estimating carriage and transmission characteristics of *S. pneumoniae *serotypes according to inclusion in the vaccine.

## Methods

### Data

A five month longitudinal study of 3–6-year-old children in 81 schools, presented in detail elsewhere [15], was conducted from January to May 2000 in France. Oropharyngeal pneumococcal colonization was monitored. Swabs were collected in the schools approximately every month, for a five month period. The mean time lag between consecutive swabs was 37 days (standard deviation: 15 days). During the observation period, 9,857 swabs were collected for serotyping. Among the 4,488 3–6-year-old children attending the schools (88% of the 3–6-year-old children in the area under study), 2,445 (55%) gave at least one swab. Among children giving at least one swab, the mean number of swabs was four (min-max: one – five). All children attending the schools were included in the analysis, even those without a single observation during the follow-up.

The analysis was restricted to the carriage of the 16 serotypes that were isolated in at least 30 swabs in the selected schools. Table 1 presents the results of the serotyping.

**Table 1:** Results of serotyping during follow-up of 4,488 children in 81 schools, January-May 2000, France.

		Frequency (%)
Serotypes with at least 30 swabs		
Vaccine serotypes†	19F	403 (4.1)
	6B	313 (3.2)
	23F	274 (2.8)
	14	191 (1.9)
	18C	79 (0.8)
	9V	79 (0.8)
Non-vaccine serotypes	6A	281 (2.9)
	3	238 (2.4)
	19A	212 (2.2)
	11A	80 (0.8)
	15A	72 (0.7)
	15B	54 (0.5)
	23A	46 (0.5)
	17F	44 (0.4)
	10A	42 (0.4)
	9L	32 (0.3)
Other serotypes		247 (2.5)
Non-colonized		7,170 (72.7)
Total		9,857 (100.0)

† Serotype included in the 7-valent conjugate vaccine.

The only currently available pneumococcal conjugate vaccine is the seven-valent vaccine, which includes serotypes 4, 6B, 9V, 14, 18C, 19F and 23F. Hence, we divided the serotypes selected for analysis into two groups: vaccine serotypes, and non-vaccine serotypes. The two groups are detailed in table 1. Vaccine serotype 4 was not selected for analysis because it was isolated in 10 swabs only. At the time of the study, conjugate vaccine had not been introduced in France, so that children participating in the study were all unvaccinated.

**Figure 1:**
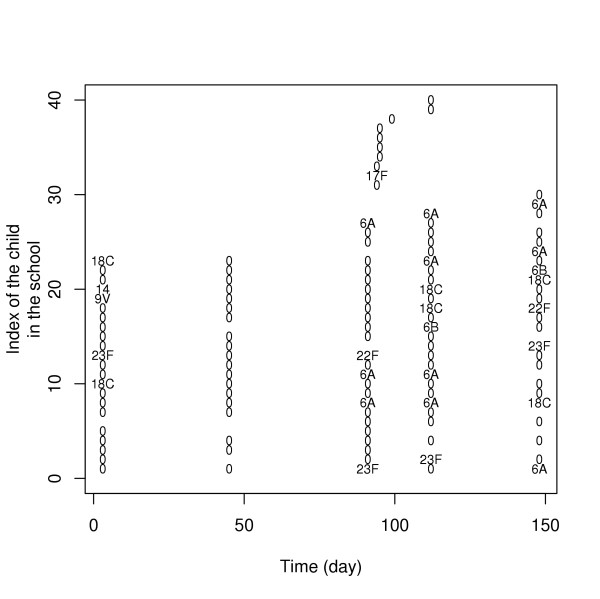
**Swabs collected in a school participating in the study**. "0" indicates that the sample was taken but no serotype was detected; otherwise, serotype number is given.

Figure 1 presents data collected in a school participating in the study. Among the 41 children of the school, 15, 6, 3, 4, 12 and 1 children gave respectively 5, 4, 3, 1 and 0 swabs. While serotype 6A was not detected in the school at the beginning of the follow-up, a micro-epidemic was observed during the second half of the follow-up, with at least 8 children carrying serotype 6A during this time period.

### Transmission model

Here, we present a dynamic model for *S. pneumoniae *transmission in schools. The model detailed the individual rate of colonization and decolonization by *S. pneumoniae *for all children attending the schools (even those that gave no swab at all). In the subsequent section "Estimation of transmission parameters", we show how model parameters may be estimated from the data.

In the model, we assumed that dual colonization was impossible (a child colonized by one serotype may not acquire another unless first clearing). Non-colonized children could be colonized within their school (see § *Within-school acquisition*), or in the community (*i.e*. out of the school, see § *Other assumptions*).

### Duration of carriage

We assumed that the duration of carriage of a given serotype had an Exponential distribution with mean *μ*_*V *_for vaccine serotypes and *μ*_*U *_for non-vaccine serotypes. This particular distribution is in good agreement with observed data [10].

### Within-school acquisition

We considered the school as a dynamic environment, i.e the number *C*_*s*_(*t*) of children colonized by serotype *s *at time *t *was a function of time. We assumed that, for each child who was not colonized at time *t*, the individual rate to acquire serotype *s *in the school at time *t *was i) proportional to *C*_*s*_(*t*), since this increased opportunities for transmission; and ii) inversely proportional to the size *n *of the school to allow for reduced frequency of contacts in each pair of children in larger schools [16]. With these assumptions, the *individual *rate to acquire serotype *s *at time *t *was *β C*_*s*_*(t)/n*, where *n *was the size of the school (including children that gave no swab at all) and *β *corresponded to pairwise child-to-child transmission rate, irrespective of the size *n *of the school. Note that this formulation leads to an aggregate rate of colonization due to intra-school transmission of *β C*_*s*_*(t) S(t)/n*, where *S(t) *is the number of non colonized children, in agreement with the standard Susceptible-Infectious-Susceptible model [17]. We denoted the child to child transmission rate *β*_*V *_for vaccine serotypes, and *β*_*U *_for non-vaccine serotypes.

### Other assumptions

We assumed that, during the 5-months follow-up, a non-colonized child was exposed to a rate *α*_*s *_to acquire serotype *s *in the community, with *α*_*s *_constant over time. It was also necessary to define the probability that a child carried serotype *s *at the beginning of the follow-up by *π*_*s*_. The values of *α*_*s *_and *π*_*s *_were serotype dependent. Parameters *α*_*s *_and *π*_*s *_will be considered according to two clusters to be learnt from the data through the model.

### Characterizing transmission from the model

The model was used to calculate the monthly probability to acquire a serotype in the community, which is *1-exp(-α*_*s*_*30) *for serotype *s*. It was also possible to calculate the Secondary Attack Rate (*SAR*), defined here as the probability that a colonized child transmits the bacteria to a non-colonized child of his/her school for a colonization event. The Secondary Attack Rate combines information on the mean duration of carriage *μ *and the child to child transmission rate *β*, and therefore allowed investigation of whether vaccine serotypes had 'globally' a better fitness for transmission than non-vaccine serotypes. Assuming that there is no transmission beyond the secondary case and that there is at maximum one acquisition of carriage per susceptible during the one month period, the probability of transmission between two children of a school of size *n *is:

SAR=∫0∞(1−exp⁡(−βL/n))f(L)dL
 MathType@MTEF@5@5@+=feaafiart1ev1aaatCvAUfKttLearuWrP9MDH5MBPbIqV92AaeXatLxBI9gBaebbnrfifHhDYfgasaacH8akY=wiFfYdH8Gipec8Eeeu0xXdbba9frFj0=OqFfea0dXdd9vqai=hGuQ8kuc9pgc9s8qqaq=dirpe0xb9q8qiLsFr0=vr0=vr0dc8meaabaqaciaacaGaaeqabaqabeGadaaakeaacqWGtbWucqWGbbqqcqWGsbGucqGH9aqpdaWdXaqaamaabmaabaGaeGymaeJaeyOeI0IagiyzauMaeiiEaGNaeiiCaaNaeiikaGIaeyOeI0ccciGae8NSdigcbiGae4htaWecbaGae03la8Iae4NBa4Mae0xkaKcacaGLOaGaayzkaaGaemOzayMaeiikaGIaemitaWKaeiykaKIaemizaqMaemitaWealeaacqaIWaamaeaacqGHEisPa0Gaey4kIipaaaa@4B88@

where *L *is the duration of carriage with density *f(L)=exp(-L/μ)/μ *and *1 *- *exp(- β L/n) *is the probability that a child colonized during time period *L *transmits to a non-colonized child of the school. Eventually, the Secondary Attack Rate was equal to (*1+Nμ *^-1 ^*β*^-1^)^-1 ^within a school of size *n*.

### Estimation of transmission parameters

#### Data augmentation

**Figure 2:**
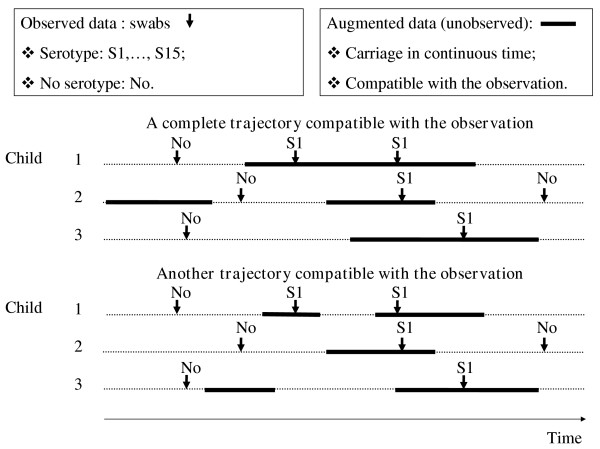
**Data augmentation strategy to estimate transmission parameters of *S. pneumoniae *from the longitudinal follow-up of pneumococcal carriage in schools**. The observed data consist of the times when swabs are collected in the school. The data are augmented with the times of colonization/decolonization. In the MCMC algorithm, augmented periods of carriage may be added/suppressed, split/combined; and the times of colonization/decolonization may change.

The transmission model may easily be estimated with likelihood-based approaches from complete data that consist of the times of colonization and decolonization for each child. In practice however, carriage is only observed at few times, so that the likelihood of the data is not readily available. Figure 2 illustrates the data augmentation strategy adopted to circumvent this difficulty. It consists of augmenting the data with a description of colonization in continuous time that is compatible with the observations [13].

Conditional on these augmented times and model parameters, the likelihood of the data is available, but since there is no unique way to choose the augmented times given the observation, a systematic exploration of the augmented times is necessary for inference. This is performed by Markov chain Monte Carlo (MCMC) sampling. In the algorithm, augmented periods of carriage may be added/suppressed, split/combined; and the times of colonization/decolonization may change [13]. Figure 2 presents two augmented trajectories that are compatible with the observation and could be explored by our algorithm.

#### Heterogeneity in the community acquisition rates of serotypes and in the probabilities of carriage at the beginning of follow-up

Heterogeneity was allowed in the community acquisition rates of serotypes and in the probabilities of carriage at the beginning of follow-up. More precisely, we allowed serotypes to cluster in two subgroups, not fixed in advance, for each of these parameters. In the MCMC sampling scheme, changes in serotypes allocation were proposed independently for community acquisition rate and probability of carriage at the beginning of the follow-up.

#### Bayesian hierarchical framework

The statistical framework has a Bayesian hierarchical structure [18], with 3 levels:

A) The observation level ensures that the augmented data are consistent with the observation;

B) The transmission level describes the latent transmission process (transmission model);

C) The *prior *level specifies the *prior *distributions of the parameters: For the child to child transmission rate *β*, the community acquisition rate *α *and the mean duration of carriage *μ*, we specified vague flat priors consisting of Exponential distributions with means 10^5 ^day^-1^, 10^5 ^day^-1 ^and 10^5 ^days, respectively. The *prior *distribution for the probability *π *of carriage at the beginning of the follow-up was uniform from 0 to 1.

### MCMC implementation

The MCMC algorithm was developed in C; the output was analyzed with R software. The seed used in the simulations was given by the computer clock. We performed 1,000,000 iterations for each run of the MCMC algorithm. The first 500,000 were discarded as the burn-in period. The output was then recorded once every 5 iterations to constitute a sample from the posterior distribution. The convergence was visually assessed, and tested with the Geweke criteria [19]. We also checked that estimates were robust to a change in the initial values.

Eventually, the joint *posterior *distribution of augmented data, clusters of serotypes and parameters was explored by MCMC sampling, and characterized by means and equal-tailed 95% credible intervals (CI). For the community acquisition rates and the probabilities of carriage at the beginning of the follow-up, reported results correspond to the partition of serotypes with the largest *posterior *probability.

## Results

Table 2 gives the *posterior *mean (95% CI) of carriage and transmission parameters of *S. pneumoniae*.

**Table 2:** *Posterior *mean and 95% credible interval of transmission parameters of *S. pneumoniae *according to serotype inclusion in the vaccine. Transmission parameters consist of the mean duration of carriage *μ*, the child to child transmission rate *β *and the Secondary Attack Rate (*SAR*).

	Vaccine serotype*		Non-vaccine serotype†		Ratio	
	Mean	95% CI‡	Mean	95% CI‡	Mean	95% CI‡

*μ *(days)	23	21, 25	22	20, 24	1.06	0.94, 1.18
*β *(% day^-1^)	4.6	4.2, 5.0	5.1	4.5, 5.6	0.91	0.80, 1.05
*SAR *(%)						
*n *= 30 §	3.4	3.2, 3.7	3.6	3.3, 3.8	0.97	0.88, 1.06
*n *= 50 §	2.1	1.9, 2.2	2.2	2.0, 2.3	0.97	0.88, 1.06
*n *= 100 §	1.1	1.0, 1.2	1.1	1.0, 1.2	0.97	0.88, 1.06

* Serotypes 6B, 9V, 14, 18C, 19F, 23F which are included in the 7-valent conjugate vaccine were selected for analysis (at least 30 positive swabs). Vaccine serotype 4 was not selected for analysis because it was isolated in 10 swabs only.

† Serotypes 6A, 3, 19A, 11A, 15A, 15B, 23A, 17F, 10A, 9L were selected for analysis.

‡ CI, credible interval.

§*n*, size of the school.

### Durations of carriage

The mean duration of carriage was *μ*_*V *_= 23 (95% CI: 21, 25) days for vaccine serotypes and *μ*_*U *_= 22 (95% CI: 20, 24) days for non-vaccine serotypes, leading to a relative mean duration of carriage (*μ*_*V*_*/μ*_*U*_) equal to 1.06 (95% CI: 0.94, 1.18).

### Colonization rate within a school

The child to child transmission rate was *β*_*V *_= 0.046 (95% CI: 0.042, 0.050) day^-1 ^for vaccine serotypes and *β*_*U *_= 0.051 (95% CI: 0.045, 0.056) day^-1 ^for non-vaccine serotypes, leading to a relative child to child transmission rate (*β*_*V*_*/β*_*U*_) equal to 0.91 (95% CI: 0.80, 1.05). In schools of size 30, 50, and 100, the Secondary Attack Rate was respectively 3.4% (95% CI: 3.2%, 3.7%), 2.1% (95% CI: 1.9%, 2.2%) and 1.1% (95% CI: 1.0%, 1.2%) for vaccine serotypes, and 3.6% (95% CI: 3.3%, 3.8%), 2.2% (95% CI: 2.0%, 2.3%) and 1.1% (95% CI: 1.0%, 1.2%) for non-vaccine serotypes. The relative Secondary Attack Rate (*SAR*_*V*_*/SAR*_*U*_) was 0.97 (95% CI: 0.88, 1.06) irrespective of the size of the school.

### Other parameters

The *posterior *probability of the partition with largest support was 84% for the community acquisition rate and 92% for the probability of carriage at the beginning of the follow-up. Serotypes clustered identically regarding community acquisition rate and probability of carriage at the beginning of the follow-up. The first cluster of serotypes contained 9 serotypes (23A, 17F, 10A, 15B, 9V, 11A, 18C, 15A, 9L). It was characterized by a monthly probability of colonization in the community estimated at 0.40% (95% CI: 0.32%, 0.47%) per serotype, and a probability of carriage at the beginning of the follow-up equal to 0.48% (95% CI: 0.40%, 0.57%) per serotype. The second cluster consisted of 7 serotypes (19A, 14, 3, 6A, 23F, 6B, 19F). The monthly probability to acquire one of these serotypes out of the school was 1.40% (95% CI: 1.20%, 1.65%) per serotype, with a probability of carriage at the beginning of the follow-up equal to 2.3% (95% CI: 2.1%, 2.5%) per serotype. Serotypes that were found to have high community acquisition rate/probability of carriage at the beginning of the follow-up were those that were the most prevalent in the schools (191–403 swabs, as opposed to 32–80 swabs for other serotypes).

## Discussion

We found that, in 3–6 years old children, the mean duration of carriage and the child to child transmission rate of *S. pneumoniae *was the same for vaccine and non-vaccine serotypes, from the analysis of a large cohort of schoolchildren.

Carriage was determined by oropharyngeal swabbing at each visit. This procedure is known to be less sensitive than nasopharyngeal swabbing [20]. Therefore, some samples could be falsely negative, and lead to underestimate the duration of carriage. However, our estimates of the mean durations of carriage (about 20 days for 3–6 years old children) are consistent with those reported by Ekdahl et al. [10]. Melegaro et al. [12] also found a carriage duration of 20 days for children ≥ 5 years old, but of 50 days for children <5 years old. This difference could be explained by the presence of children under 2 years old, who are known to have the largest durations of carriage [10] and were not covered by our study, and also by a coarser characterization of serotypes in Melegaro et al. [12]. A larger estimate of the duration of carriage (45 days) was found by Auranen et al. [13] for subjects older than 2 years old. Here again, a possible difference in the age distributions of the population under study might explain the differences in the estimates.

Here, we detected no difference between vaccine and non-vaccine serotypes for durations of carriage, child to child transmission rates and Secondary Attack Rates among school age children. These results are consistent with those of Ekdahl et al. [10] who found no significant difference between durations of carriage of serogroups when adjusted for age; but contrast with those of Smith et al. [11] who concluded that there was a factor of 10 between the mean durations of carriage of the most/least persistent serotypes. Swabs were collected weekly in [10]; and monthly in [11] and in our study (80% of intervals between paired nasal swab specimens were larger than ≥ 28 days in the study of Smith et al.; this proportion is even larger in our study). Our approach could be used to re-analyze the data of Smith et al [11] and investigate whether their results are robust to: i) the possible occurrence of decolonization/re-colonization between consecutive positive swabs; ii) the inclusion of micro-epidemics in the stochastic transmission model. Since sensitivity of oropharyngeal swabbing is not likely to be serotype-dependent, differences between vaccine and non-vaccine durations of carriage should not be affected if durations of carriage were underestimated. Simulation studies showed that we could have detected differences between durations of carriage of the order of one week, but also that the sampling interval (approximately 1 month in our study) was sufficient to estimate durations of carriage in the considered range (≥18 days). It would be important to investigate if it is still the case with shorter durations of carriage.

**Figure 3:**
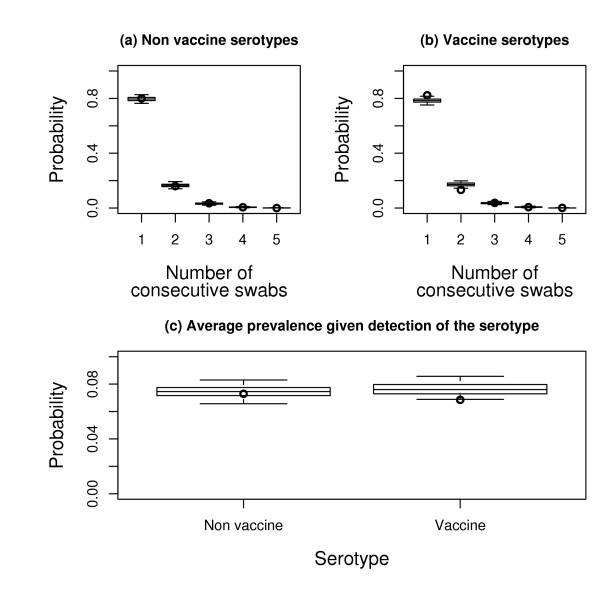
**Posterior predictive check**. Panel a and b: Predictive distribution of the number of consecutive swabs in which the same serotype was detected for non-vaccine (panel a) and vaccine (panel b) serotypes. Panel c: predictive distribution of the serotype-specific average prevalence given detection of the serotype in the school. Boxplots give quantiles 2.5%, 25%, 50%, 75% and 97.5% of the distributions; circles indicate observed values. The predictive distributions are derived from 700 epidemics simulated with transmission parameters drawn from the *posterior *distribution.

Simple statistics from the raw data supported that child to child transmission rates and mean durations of carriage were similar across vaccine and non vaccine serotypes. Figure 3 shows the distribution of the number of consecutive swabs in which the same serotype was detected in the same children. This distribution was the same for non-vaccine (circle, panel a) and vaccine (circle, panel b) serotypes, suggesting similar durations of carriage since the frequency of sampling was not serotype dependent. We also calculated, for each school and each month during the follow-up, the serotype-specific prevalence as the ratio (number of positive swabs for serotype *s*)/(total number of swabs) for serotype *s*. The average prevalence was expectedly larger for vaccine serotypes (2.1%) than for non vaccine serotypes (1.1%). But once a serotype was detected in the school, its average prevalence was 7% irrespective of inclusion in the vaccine. This suggests that inter-individual transmission in schools was similar for vaccine and non-vaccine serotypes. Next, we performed *posterior *predictive check using simulations from the transmission model used for analysis (see the Appendix). Figure 3 gives the distribution of the number of consecutive swabs predicted by the model for non-vaccine (boxplot, panel a) and vaccine (boxplot, panel b) serotypes, and the predictive distribution of prevalence given detection of the serotype (boxplot, panel c). For the 2 criteria, related respectively to duration of carriage and to micro-epidemics (~ child to child transmission rate), model predictions closely match the data.

Infants are known to be the main reservoir in maintaining transmission. Consequently, the differences we found in the community acquisition rates could proceed from heterogeneity in serotype-specific prevalence in infants. Our data does not allow to judge whether serotype-specific prevalence in infants could be compatible with the absence of differences in the duration of carriage and in the child to child transmission rate (like it is for 3–6 years old children).

While the absence of difference between vaccine and non-vaccine serotypes seems at odds with the large differences observed in serotypes prevalence, mathematical models have shown that, in the context of direct competition with short term immunity [6-9] or in the context of indirect competition with long term immunity [6], the difference in serotypes prevalence could be much larger than what would be expected in the absence of competition. For example, in [6], competition between 2 serotypes with close transmission characteristics (basic reproduction number 2.2 and 1.8 respectively) led to extinction of the serotype with reduced transmissibility. Temime et al. [9] have shown that competition between serotypes was sufficient to generate large differences in prevalence according to serotypes, even in the absence of difference in transmission characteristics. While different modes of competition may influence serotypes equilibrium prevalence, replacement may occur only in case of direct competition in the absence of long-term immunity [6]. In this context, the extent of replacement depends on the degree of competition, but also on serotype specific transmission characteristics, which determine the ability of non vaccine serotypes to re-colonize the ecological niche released by vaccine serotypes.

In order to explain differences in the prevalence of carriage, heterogeneity is required in at least one parameter among the duration of carriage, the child to child transmission rate and the community acquisition rate. In this respect, if duration of carriage and child to child transmission rate had been fixed to the same value for all serotypes, differences in the community acquisition rate would have naturally shown to match the differences in serotype specific prevalence in the data. However, our purpose was here to determine whether the data could indicate which differences were the most likely. To that end, we imposed that the mean duration and the child to child transmission rate should be the same among vaccine serotypes and among non vaccine serotypes, but allowed for differences between these two classes. In this framework, it is possible to obtain differences in the mean duration of carriage, or child to child transmission rate, between vaccine and non-vaccine serotypes, irrespective of a difference in the community acquisition rate. We have found, using simulations, that the data and procedure were informative and selective enough to do this: changes in the mean duration of carriage, in the presence of heterogeneity in the community acquisition rate, were identifiable in the framework described.

In this paper, we focused on pneumococcal carriage and not on pneumococcal acute otitis media or invasive disease. While our results favour the hypothesis that replacement should follow vaccination, the overall impact on pneumococcal disease is less clear. In a vaccinated population the othopathogenic capacities of the replacing non-conjugate vaccine type pneumococci have been established [21,22]. For invasive diseases, some serotypes not included in the vaccine appear to be less invasive [14] and a reduction in incidence could follow the introduction of the vaccine despite replacement in carriage.

We considered a model in which competition was direct and prevented dual colonization. This last assumption is strong since dual colonization is possible [23,24]. However, given the nature of our data in which at most 1 serotype may be detected per swab, this was the only sensible approach. If information on dual colonization were available in the data, the approach could easily be extended to estimate competition parameters as listed by Lipsitch [7]. Our approach, similar to that of Auranen et al. [13], amounted to consider that in case of dual colonization in one individual there was a "major" serotype that would be transmitted. The results should be robust to this assumption unless there was a significant transmission of "minor" serotypes in case of dual transmission.

In our analysis, we investigated transmission of the 16 serotypes that were the most prevalent in the dataset. We assumed that individuals carrying less prevalent serotypes were susceptible to colonization by serotypes under study (absence of direct competition). This may have an effect on the duration of carriage estimates as out competition may have occurred rather than clearance of the serotype that was originally carried. However, the effect should be limited since serotypes included in the analysis represent 93% of overall carriage.

Heterogeneity in community acquisition rates and probabilities of carriage at the beginning of the follow-up was explored by allowing free cluster formation according to these parameters. The number of clusters was fixed (= 2), but the composition of the clusters was determined by the MCMC algorithm. With a number of clusters fixed to 3 or 4, the estimates of the mean durations of carriage and the child to child transmission rates remained unchanged.

## Conclusion

In conclusion, this study supports that, in 3–6 years old children, no competitive advantage exists for vaccine serotypes compared to non-vaccine serotypes. This is an argument in favour of important serotype replacement. It would be important to validate the result for infants, who are known to be the main reservoir in maintaining transmission. Overall reduction in pathogenicity should also be taken into account in forecasting the future burden of pneumococcal colonization in vaccinated populations.

## Abbreviations

CI, Credible Interval;

*SAR*, Secondary Attack Rate;

*S. pneumoniae*, Streptococcus pneumoniae;

MCMC, Markov chain Monte Carlo.

## Appendix: Simulations

Seven hundred epidemics were simulated from the model, with transmission parameters for the serotypes (child to child transmission rate *β*, community acquisition rate *α *and mean duration of carriage *μ *for each serotype) drawn from the *posterior *distribution. The probability of carriage at time 0 was not sampled from the *posterior *distribution. Rather, we let the simulation start in the remote past (t = -1000) with no carriage in the school. The distribution of carriage at time 0 is therefore only dependent on the transmission parameters *β*, *α *and *μ*. The simulations were designed so that field and simulated data had the same structure: same number of schools and same number of children per school; same number of swabs for children *j *of schools *i *of both data, collected at the same times.

## Competing interests

The author(s) declare that they have no competing interests.

## Authors' contributions

SC conceived the study, developed and carried out statistical analyses and drafted the manuscript. LT conceived the study and helped drafting the manuscript. AJV helped drafting the manuscript. EV carried out serotyping. GT participated in the development of statistical methods for data analysis. DG conceived the study, provided the data from a field trial and helped drafting the manuscript. PYB conceived the study and helped drafting the manuscript.

## Pre-publication history

The pre-publication history for this paper can be accessed here:


